# Electrochemical Performance of Dysprosium-Doped Strontium Cobaltite with Perovskite Structure

**DOI:** 10.3390/molecules30224437

**Published:** 2025-11-17

**Authors:** Sergey N. Vereshchagin, Vyacheslav A. Dudnikov, Sergey M. Zharkov, Galina M. Zeer, Leonid A. Solovyov

**Affiliations:** 1 Institute of Chemistry and Chemical Technology SB RAS, Federal Research Center “Krasnoyarsk Scientific Center SB RAS”, Akademgorodok 50/24, 660036 Krasnoyarsk, Russia; leosol@icct.ru; 2 Siberian Federal University, 660041 Krasnoyarsk, Russia; zharkov@iph.krasn.ru (S.M.Z.); gzeer@sfu-kras.ru (G.M.Z.); 3 Kirensky Institute of Physics, Federal Research Center KSC SB RAS, Akademgorodok 50/38, 660036 Krasnoyarsk, Russia; slad63@yandex.ru

**Keywords:** perovskite, cobaltite, supercapacitors, rare earth oxides

## Abstract

The electrochemical properties of the dysprosium-doped strontium cobaltite SDC (Sr_0.8_Dy_0.2_CoO_3−δ_) were evaluated for possible application as pseudocapacitor electrode materials. Dense perovskite SDC ceramics were prepared using standard solid-state synthesis techniques. The SDC sample was characterized using XRD, structural analysis, SEM/EDS, and simultaneous thermal analysis. The electrochemical performance of the electrode was estimated in 3M KOH in a standard electrochemical cell for corrosion studies using cyclic voltammetry, impedance spectra, galvanostatic charge/discharge, and long-term cycling stability. The study demonstrated that the SDC exhibits high oxygen mobility and has the ability to release or incorporate oxygen from the gas phase. This process leads to the formation of structural anion vacancies without compromising the structural integrity. The SDC electrode demonstrates a specific capacitance of approximately 500 F/m^2^ and exhibits satisfactory cyclability. Electrochemical treatment in charge–discharge cycles has been shown to result in the formation of a thin strontium-depleted layer on the electrode surface. The observed behavior is believed to be caused by a high concentration of oxygen vacancies, which is consistent with oxygen intercalation into the perovskite structure. The present study suggests that rare earth-doped strontium cobaltite may serve as a prospective precursor for electrode material in supercapacitors.

## 1. Introduction

Oxides with the perovskite structure (ABO_3_) are promising systems for creating materials for energy production [1,2,3] and storage [4]. Among these materials, supercapacitors are currently attracting increasing attention due to their high energy density, power, charge–discharge rate, and long-term cyclic stability. Supercapacitors are classified into three types, depending on the material used and the energy storage mechanism: (i) electric double layer capacitors, (ii) pseudocapacitors, in which a faradaic redox reaction plays a decisive role in charge storage, and (iii) hybrid supercapacitors, which include a capacitor electrode as the anode and a battery-like electrode as the cathode [5].

The implementation of pseudocapacitor capacitance is believed to occur through three mechanisms [6]: (i) the precipitation (adsorption-desorption) of ions at a potential below that expected for the redox reaction, (ii) the reversible, surface-based, Faradaic reactions of active substances (e.g., transition metal oxides and conducting polymers), and (iii) the reversible intercalation of ions from the electrolyte into the crystalline structure of the electrode material. When intercalation pseudocapacitance occurs, the charge storage region expands into the volume of the electrode material, not just its surface. Thus, the implementation of this mechanism leads to a significant increase in the energy density of supercapacitors [7]. The most important prerequisites for intercalation pseudocapacitance are pathways that allow for the rapid diffusion of intercalated ions within the crystalline structure, as well as the ability to accommodate sufficient quantities of electrolyte ions without phase changes of the material.

Promising materials for implementing this type of pseudocapacitance include phases that can intercalate O^2−^ ions. In this case, the amount of charge retained during one charge/discharge cycle doubles compared to the use of singly charged ions (e.g., Li^+^). It is believed that, in an alkaline electrolyte (NaOH or KOH), oxygen intercalation involves the diffusion of the OH^−^ ion from the crystal surface into the bulk through oxygen vacancies and the oxidation of the transition metal cation located in the B-sublattice of the perovskite structure, which occurs simultaneously with the formation of water [8]. Thus, promising materials for intercalation pseudocapacitance based on O^2−^ ions should be able to vary the concentration of oxygen vacancies over a wide range while remaining structurally stable during the removal or incorporation of oxygen. Materials based on Sr, La, Ce, Y, Pr, Gd, Ba, Ni, Co, or Fe with a perovskite structure or a Ruddlesden-Popper phase A_2_BO_4_ that satisfy this condition have shown high values of specific capacity: 1222 F/g (NiO/LiNO_3_) [9], 1050 F/g (Ba_2_Bi_0.1_Sc_0.2_Co_1.7_O_6−δ_) [10], and 983.6 F/g (La_0.7_Sr_1.2_Fe_0.9_Co_0.1_O_4_) [11].

Previous studies have shown that rare-earth metal-doped, non-stoichiometric strontium perovskites Sr_x_Ln_1−x_CoO_3−δ_, as well as the Ruddlesden-Popper Sr_2.4_Ln_0.6_Co_2_O_7−δ_ phases (Ln = Gd, Dy), have a high concentration of oxygen vacancies and oxygen/(oxygen vacancy) mobility. These materials also demonstrate structural stability over a wide range of δ [12,13,14]. Specifically, we found that RP compounds can withstand oxygen removal up to δ = 1 while maintaining the crystal structure of the parent A_3_Co_2_O_7_ phase (A = Sr_2.4_Ln_0.6_).

This study aimed to investigate the electrochemical performance of dysprosium-strontium cobaltite to evaluate the potential use of perovskite SrCoO_3_ doped with rare earth cations as an electrode material based on the concept of intercalation pseudocapacitance involving O^2−^ ions.

## 2. Results and Discussion

### 2.1. Structural and Functional Analysis

**Figure 1:**
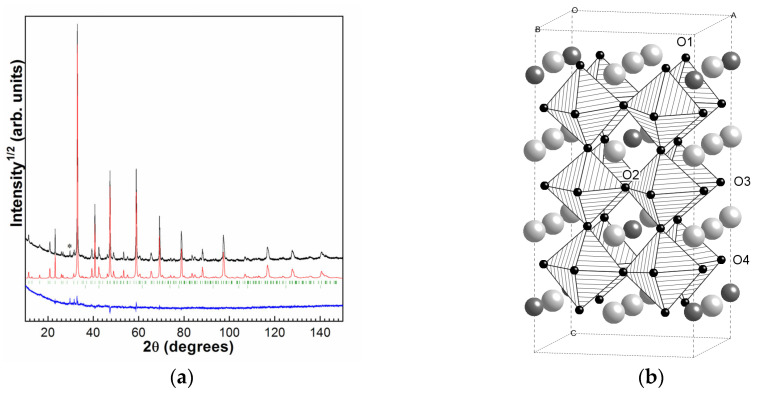
(**a**) Room temperature X-ray diffraction patterns after crystal structure refinement for the ordered Sr_0.8_Dy_0.2_CoO_2.64_: black—experimental, red—calculated, and blue difference curves. The calculated reflection positions of the tetragonal phase and cobalt oxide are shown by strokes. (**b**) Structure of ordered tetragonal Sr_0.8_Dy_0.2_CoO_3−δ_. Gray octahedra represent Co^n+^, gray spheres represent Sr^2+^, dark gray spheres represent Dy^3+^/Sr^2+^, and smaller black spheres are oxygen (the O2 site shows the predominant O vacancy location). The asterisk marks the Cu K_β_ line of the main reflection.

The synthesized SDC after slow cooling in air has a stoichiometry of Sr_0.8_Dy_0.2_CoO_2.65_. The PXRD pattern of the SDC sample is presented in Figure 1a. In addition to the main tetragonal phase of SDC, about 0.5% of cobalt oxide was found in the synthesized ceramics. According to the full-profile structure refinement, the structure of SDC corresponds to the tetragonal *I4/mmm* superstructure (*a* = 7.6716(3) Å, *c* = 15.3659(9) Å) with ordered anion vacancies and Dy^3+^/Sr^2+^ cations. The structure is identical to that described in [15], observed earlier by us for Sr_0.8_Gd_0.2_CoO_3−δ_ [16] and for Sr_0.8_Dy_0.2_CoO_3−δ_ [17]. The most important feature of the structure is that in addition to the Dy^3+^/Sr^2+^ cations ordering in three distinct crystallographic A-positions, it accommodates four positions for O^2−^ anions which is also the sites for anion vacancies. The regular arrangement of Sr^2+^/Dy^3+^ positions are accompanied by orderliness in the oxygen vacancies’ location (which are situated mostly in O2 sites) [15], Figure 1b.

As noted in the introduction, the presence of oxygen vacancies can favorably affect the system’s ability to realize intercalation pseudocapacity. In the tetragonal and cubic phases of Sr_x_La_(1−x)_CoO_3−δ_, the presence of anion vacancies is related to the nonstoichiometry coefficient δ, and their number can be controlled by oxygen addition/abstraction processes. It has been established that under conditions of heating, strontium cobaltites doped with rare earth cations exhibit the reversible loss of oxygen [18,19] according to Equations (1) and (2), written as follows:

(1) 2 Co^′^_Co_ + V^••^_O_ + 1/2 O_2_ ↔ 2 Co^x^_Co_ + O^x^_O_

(2) 2 Co^x^_Co_ + V^••^_O_ + 1/2 O_2_ ↔ 2 Co^•^_Co_ + O^x^_O_

**Figure 2:**
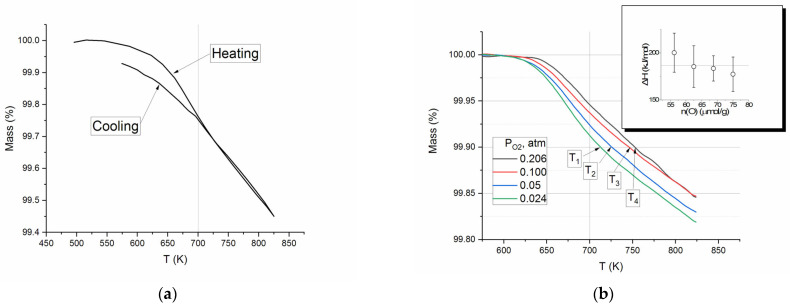
(**a**) Typical TG curve of SDC sample in the heating–cooling cycle; (**b**) Change in the mass of SDC sample upon heating at β = 10°/min in O_2_-Ar atmosphere with different partial pressures of oxygen. The gray lines denote equal values of mass loss Δm. T_1_–T_4_ are the temperatures for Δm = 0.1%.

The amount of oxygen removed and the rate of the process depend on temperature, oxygen partial pressure, composition x, and the initial value of the non-stoichiometry *coefficient* δ of cobaltite Sr_x_Ln_(1−x)_CoO_3−δ_. The onset of this process is typically observed at temperatures in the range of 550–600 K. The oxygen evolution can be monitored by TG analysis (Figure 2a), which involves measuring mass loss during heating and cooling cycles. Figure 2a shows mass variation in a single cycle of the SDC heating–cooling loop. The substantial decrease in mass begins at approximately 525 K and declines at T > 650 K almost linearly with increasing temperature up to 825 K. The cooling curve indicates an increase in mass; the reverse line corresponds to the heating line up to 700 K and deviates from it at temperatures below that threshold.

The TG experiments demonstrate distinctly that at temperatures exceeding 700 K (under the given conditions: temperature ramp rate of 10°/min, thin layer of SDC particles with a size of less than 0.16 mm) the process of oxygen removal/addition can be considered quasi-equilibrium. Assuming that at T > 700 K there is an equilibrium of gas-phase oxygen and the solid phase, one can estimate the enthalpy of O_2_ removal. Figure 2b shows the TG curves for SDC heated under different partial pressures of O_2_. According to the Equation (3), the enthalpy ΔH of O_2_ removal is a slope of a line in coordinates ln(P) − ln $\begin{array}{c} f_{\left(\right)} \end{array}$ P 1/T1/T.

(3) $$\ln\left(P\right)=-\frac{∆H}{RT}$$

where P is P0, P1  the oxygen partial pressure; T is the temperature of constant mass loss ∆m; and R R is the ideal gas constant.

The inset in Figure 2b shows the result of calculation for P_O2_ = 0.024, 0.05, 0.10, 0.206 atm and values of ∆m = 0.09, 0.10, 0.11, 0.12%, which corresponds to 56–75 μmol O removal. The values obtained were found to be consistent with the established error limits, with an average value of 186 ± 9 kJ/mol.

### 2.2. Morphological Analysis of an SDC Surface

**Figure 3:**
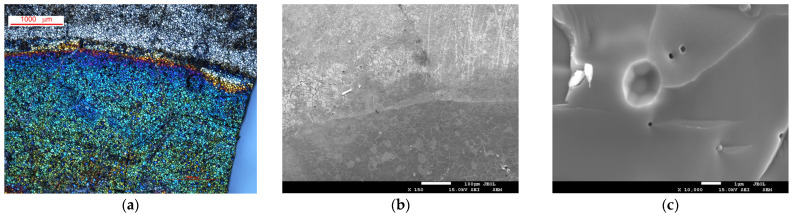
(**a**) Optical microscope image of the SDC surface; (**b**) SEM image of the SDC surface. (**c**) SEM image of the SDC electrode fracture; The upper part of the (**a**,**b**) images corresponds to the surface that was not subjected to electrochemical treatment; the lower part corresponds to the surface that was in contact with the KOH solution and was treated electrochemically. The transition zone is the area under the sealing ring.

Figure 3a,b illustrates images of the SDC electrode surface under an optical microscope and SEM, respectively. The initial surface is composed of particles measuring between 3 and 50 µm in diameter, which appear monochrome under unpolarized white light. As illustrated in Figure 3c, the image of the electrode cleavage suggests that the material is a monolithic ceramic. This ceramic is characterized by the absence of open porosity and the presence of a minimal number of individual submicron closed pores. Subsequent to the electrochemical treatment, there is a substantial alteration of the electrode surface. The appearance of colored zones on the treated part under unpolarized white light is observable through an optical microscope, corresponding to areas of varying contrast in the SEM image. The color range exhibited by the stained regions traverses a spectrum from blue to yellow, exhibiting an uneven distribution across the surface. However, the location of color areas displaying a single color tone (or contrast in SEM) corresponds with the location of grains on the original surface (Figure 3a,b). The manifestation of colored zones on the surface is most likely associated with the formation of thin surface layers (films) during processing. These layers serve as a medium for interference of white light, resulting in the creation of colorful reflections. The variation in coloration is attributed to the differential thickness of these regions. The broad distribution of colors is indicative of the corresponding distribution of film thickness around the surface.

**Figure 4:**
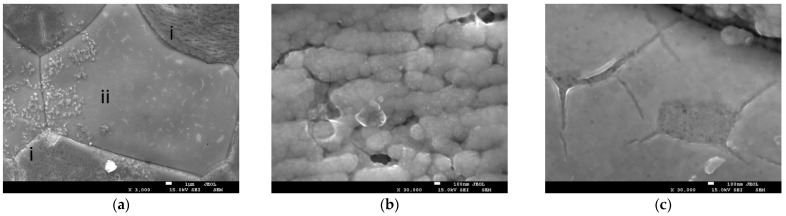
(**a**) SEM images of globular layer (i) and flat film (ii) on the SDC surface subjected to electrochemical treatment. (**b**) Enhanced image of globular layer. (**c**) Enhanced image of flat film.

An analysis of the electrode’s surface identified the presence of two distinct surface layer types, which were formed as a result of extended electrochemical exposure (Figure 4a). The globular type (see Figure 4b) was formed by globules of chain-organized round grains measuring 200–300 nm). These grains were composed of smaller nanosized particles); the estimated layer thickness was approximately 500 nm (several grains in height). The second type of film was characterized by its thin, smooth, and translucent nature, with an approximate thickness of 90 nm (Figure 4c).

The formation of layers of varying thickness and morphology, as well as their precise mapping to the grains of the solid, may be due to uneven current distribution on the surface of the ceramic tablet. In such instances, regions exhibiting elevated conductivity undergo more profound etching, resulting in accelerated coating formation in these areas. Consequently, it can be inferred that the initial stage of coating formation is the formation of a flat film, followed by the growth of individual particles and their enlargement into globules (Figure 4b).

**Figure 5:**
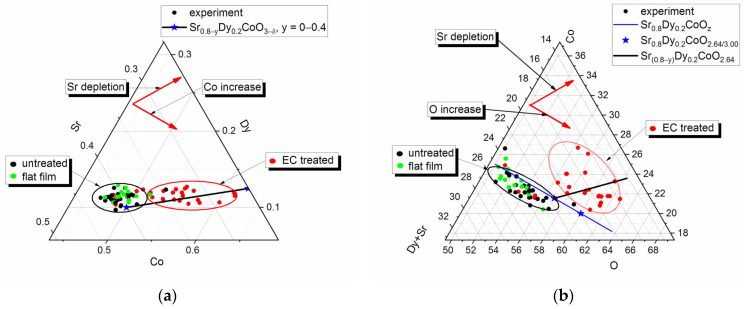
Electrode surface composition as ternary graphs. (**a**) Dy-Sr-Co; (**b**) (Sr+Dy)-Co-O. Untreated surface; electrochemically treated surface with flat film; electrochemically treated surface with globular layer; ξ composition of Sr_0.8_Dy_0.2_CoO_3−δ_ perovskite; blue and black lines show Sr_0.8_Dy_0.2_CoO_z_ (z = 2 ÷ 3.5) and Sr_0.8−y_Dy_0.2_CoO_2.64_ (y = 0 ÷ 0.4) composition, accordingly. Red arrows show trends for element content changes.

The elemental composition of the electrode interior at the fracture (Figure 3c) and its untreated surface, studied by EDS, despite significant statistical dispersion, approximately corresponded to non-stoichiometric Sr_0.8_Dy_0.2_CoO_3−δ_. The same Sr/Dy/Co/O ratio was observed for flat film domains. Statistically significant depletion in strontium and enrichment in oxygen were observed for surface areas covered by the globular layer (Figure 5a,b).

Irrespective of the nature of the surface layer, a relatively straightforward explanation for this phenomenon can be proposed. Among the three cations present (Sr^2+^, Dy^3+^, and Co^3+^), only the hydroxide Sr(OH)_2_ had limited but notable solubility in water (1.77 g/100 mL at 298 K, [20]). Consequently, only the Sr^2+^ cation can dissolve during the process of electrochemical etching. The curves (black lines) displayed in Figure 5a,b illustrate the alteration in surface composition following the extraction of Sr from the system, comprising the gross composition Sr_(0.8−y)_Dy_0.2_CoO_3−δ_ within the range of y = 0 to 0.4. These curves demonstrate a satisfactory agreement with the observed experimental trends. Similar effects were observed in the process of selective removal of Sr^2+^ by etching the surface of SrCoO_3_ while maintaining the stability of the crystal structure [21]; the selective leaching of Sr^2+^ ions into the electrolyte was also observed for a series of Ni- and Fe-containing Ruddlesden–Popper-type perovskites (La_0.125_S_r0.875_)_n+1_(Ni_0.25_Fe_0.75_)_n_O_3n+1_ (n = 1, 2, 3) under conditions of oxygen evolution reaction in water electrolysis [22].

Consequently, scanning electron microscopy analyses have revealed that during the electrochemical processing, the SDC surface undergoes transformation with the formation of a surface coating that exhibits either a smooth or a pronounced globular morphology. The surface coating features a reduced strontium content and exhibits a significantly non-uniform distribution of smooth/globular morphology. This phenomenon is likely attributable to the non-uniform distribution of electrochemical properties within the electrode volume, as well as the preferential release of strontium into the solution during the charge–discharge cycles.

### 2.3. Electrochemical Performance Analysis

**Figure 6:**
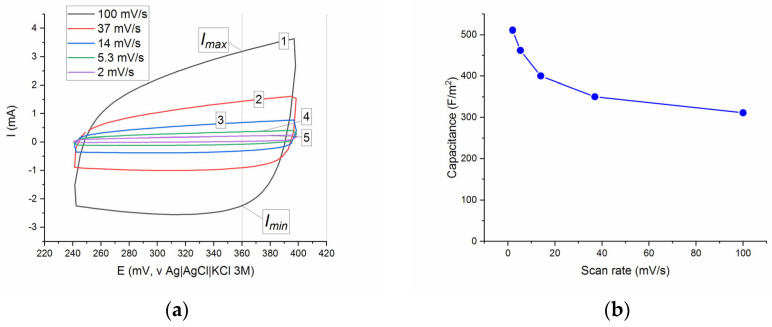
(**a**) Cyclic voltammograms of SDC at the scan rates ν of 2 (5), 5.3 (4), 14 (3), 37 (2), and 100 (1) mV/s. (**b**) Specific capacity vs. scan rate.

Figure 6a illustrates the CV curves of the SDC electrode at the scan rates ν of 2, 5.3, 14, 37, and 100 mV/s in the potential range of 0.24–0.4 V using a standard three-electrode cell system at ambient temperature. All curves had the appearance of a distorted rectangle without visible redox peaks reflecting the occurrence of oxidation–reduction processes, in contrast, in particular, to the La_0.8_Sr_1.2_Fe_0.9_Co_0.1_O_4_ system, for which extremes were observed on the anodic and cathodic CV curves, attributed to the oxidation–reduction reactions Co^2+^/Co^3+^/Co^4+^ and Fe^2+^/Fe^3+^ [11].

Since the CV curves did not show pronounced redox patterns, the values of specific capacitance C_sp_ of the SDC sample were estimated from the CV curves using simple Equation (6), as for a conventional capacitor. The C_sp_ values were calculated at E = 360 mV (vs. Ag/AgCl), and the capacity values at different scan rates are presented in Figure 6. The values of C_sp_ are in the interval of 311–511 F/m^2^, and the observed trend of C_sp_ vs. ν shows the usual gradual decrease in the specific capacity with an increase in the scan rate.

Because the values of specific capacitance published in the literature usually have the dimension of F/g, in order to compare C_sp_ obtained by us with the literature data, we converted F/m^2^ to F/g using the geometric surface area of 1 g of cubic particles with a size of 1 μm and a density of 6.13 g/cm^3^ (Sr_0.75_Dy_0.25_CoO_2.62_, ICDD # 00-056-0181). The estimated specific surface area of such particles will be 0.9788 m^2^/g. Taking into account the visible surface area of the SDC electrode (8.17 × 10^−5^ m^2^), the maximum obtained value of 500 F/g (C_sp_ = 511 F/m^2^ for scan rate ν = 2 mV/s), although inferior to the best perovskite systems [4], significantly exceeds the maximum theoretical non-Faraday capacitance of 18 μF/cm^2^ predicted in [23], which corresponds to the assumption of the participation of Faradaic processes in the formation of pseudocapacitance.

According to the classification given in [24], the observed shape of the CV curves is characteristic of materials in which the energy storage mechanism based on the formation of a double electric layer or on the reduction of surface-active components is implemented. The absence of peaks associated with the Co^2+^/Co^3+^/Co^4+^ redox transition in the studied potential range can be explained by the possibility of a monotonic change in stoichiometry in the perovskites Sr_0.8_Ln_0.2_CoO_3−δ_ or perovskite-like RP phases Sr_2.4_Ln_0.6_CoO_7−δ_ (Ln = Sm, Gd, Dy) [14]. Additional information on the nature of the processes at the electrode can be obtained from the analysis of the dependence of the current on the scan rate, expressed by Equation (4).

(4) $$I=a\nu^{b}$$

(5) ACoO_3−δ−δ1_ + 2*δ* OH^−^ ↔ ACoO_3−δ1_ + *δ* H_2_O + 2*δ e^−^*

(6) 2 Co^x^_Co_ + V^••^_O_ + 2 OH^′^ ↔ 2 Co^•^_Co_ + O^x^_O_ + H_2_O + 2 e^′^

(7) 2 Co^′^_Co_ + V^••^_O_ + 2 OH^′^ ↔ 2 Co^x^_Co_ + O^x^_O_ + H_2_O + 2 e^′^

Discrimination of the contributions of surface phenomena and diffusion within the lattice is based on an analysis of the value of b in Equation (4). The value of b in this equation is considered as an indicator of the contribution from semi-infinite linear diffusion (b = 0.5) and surface-capacitive mechanisms (b = 1).

**Figure 7:**
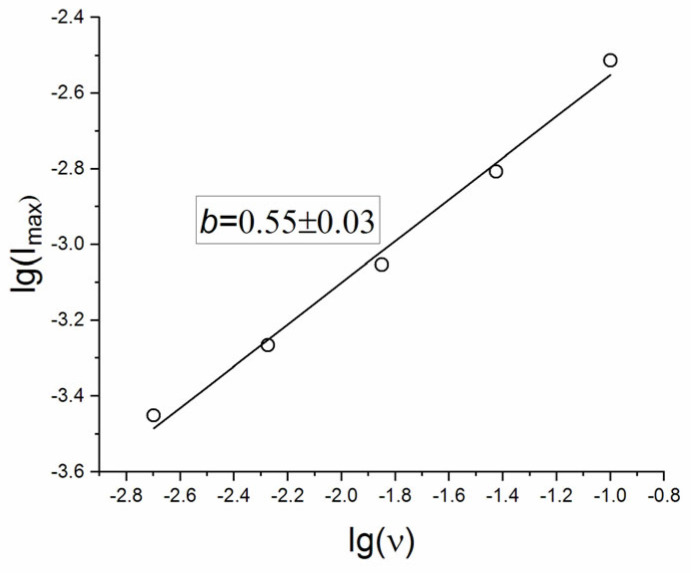
Log-log dependence of I_max_ on the potential scan rate ν.

The graph of the dependence of lg(Imax) on lg(ν) for the anodic part of the CV curve (Figure 7) shows that for SDC the b value is 0.55 ± 0.03, and therefore, one can conclude that the kinetics predominantly follows a semi-infinite linear diffusion process.

It is believed that during the intercalation process, OH^−^ anions on the perovskite surface interact with an oxygen vacancy to form water (reaction (5–7)), followed by diffusion of O^2−^ into the bulk of the electrode material; a schematic illustration of the mechanism of this oxygen–anion intercalation process using LaMnO_3_ as an example is given in [8]. It is noteworthy that the equations describing the interaction of gas-phase oxygen (1, 2) with non-stoichiometric perovskite are similar to Equations (5)–(7) in terms of the requirements for the defect structure of the solid. In both cases, the process is described as the interaction of an oxygen-containing species (O_2_/OH^−^) with an oxygen vacancy V^••^_O_ with the participation of cobalt cations Co^2+^/Co^3+^, resulting in the formation of lattice oxygen O^2−^. This similarity of the processes suggests that, despite significant differences in the process conditions (temperature range, gas/liquid phases), the presence of mobile oxygen in the system may indicate a certain potential for the emergence of pseudocapacitance based on oxygen intercalation.

**Figure 8:**
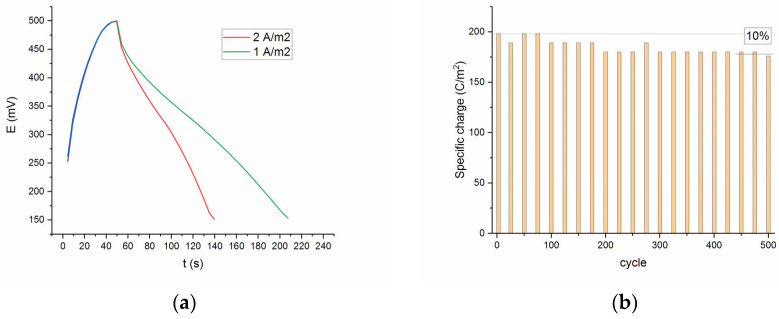
(**a**) Charge–discharge curves of SDC. Current densities: charge—5.5 A/m^2^; discharge—1 (green) and 2 (red) A/m^2^. (**b**) The cycling performance of SDC electrode, showing retention of capacitance after 500 cycles; charge/discharge current density of 2 A/g.

Figure 8a represents the discharge curves of the SDC electrode at current densities 1 and 2 A/m^2^. The discharge curves obtained for all current densities were slightly non-linear, which demonstrates the intercalation-type or intercalation-type materials showing broad but electrochemically reversible redox peaks [24]. From the discharge curve, the specific capacity *Q_sp_* was calculated using Equation (8), where the symbols *I, Δt*, and *S* represent the discharge current (A), discharge time (s) and visible surface of the electrode (m^2^), respectively.

(8) $$Q_{sp}=\frac{I\cdot ∆t}{S}$$

The specific capacity gradually decreases to approximately 90% after 500 cycles, which may be due to the leaching of strontium and the formation of a surface globular layer during electrochemical processing in an alkaline solution. As shown in Figure 8b, only about 10% capacitance loss occurred at the end of the test, indicating a satisfactory cycling performance.

**Figure 9:**
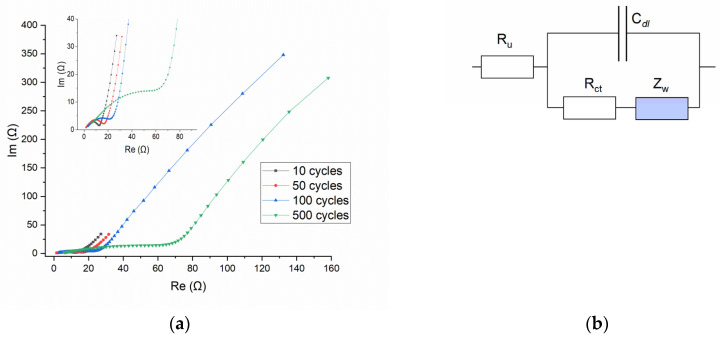
(**a**) Nyquist plots for the SDC electrode after different number of charge–discharge cycles. The inset is the expanded plots at the high frequency region. (**b**) The Randles equivalent circuit.

To better understand the electrochemical performance and dynamics of the SDC electrode, the EIS was carried out in a frequency range of 0.01–10^5^ Hz by applying an AC voltage with 10 mV amplitude. Figure 9a shows the Nyquist plots for the SDC electrode after different numbers of charge–discharge cycles, namely after 10, 50, 100, and 500 cycles; the inset is the expanded plots at the high-frequency domain. All four lines show EIS, which is typical for the so-called Randles equivalent circuit (Figure 9b) [25]. The patterns comprise the semicircle at the high-frequency region, where the electrochemical process is controlled by charge transfer phenomena, and the almost straight line at the low-frequency domain, where the electrochemical process is controlled by mass transfer phenomena. In this circuit the total current, which passes through the R_u_ (the ohmic resistance of the electrolyte between the reference and the working electrode), is a sum of the current to charge/discharge the electrical double layer (C_dl_) and the parallel current of the faradaic process. The general impedance of the faradaic process is represented by two components: the charge-transfer resistance, R_ct_, which is related to the kinetics of the heterogeneous electrochemical process, and the Warburg impedance, Z_W_, which accounts for the mass transport of the redox species to the electrode surface. Warburg impedance assumes a semi-infinite linear diffusion and is represented on the Nyquist plot by a straight line with slope 1 (φ = 45°). It is obvious that a simple Randles circuit cannot quantitatively describe the course of the EIS curves, since the slope of the low-frequency line is different from unity. But this circuit can still be used to follow the main peculiarities of the process.

From the data presented in Figure 9a, several features emerge that characterize changes in the appearance of the EIS with an increase in the number of charge–discharge cycles.

(1) The straight line at the low-frequency domain inclines at an angle considerably higher than 45°, which is indicative of deviation from ideal Warburg behavior. To model a tilted line in the low-frequency domain, a constant phase element (CPE) is used instead of an ideal capacitor. The CPE impedance includes two parameters: Y_o_, which contains information about the capacitance, and an exponent n (in the range from 0 to 1), which determines the deviation from the ideal behavior. For n = 1 CPE functions as an ideal capacitor; for n = 0 the CPE behaves as a resistor. The divergence of the impedimetric line from vertical (the angle θ; °) related to n as θ = 90°(1−n). A rough estimation of n for lines in Figure 9a gives a value of about 0.8. The physical meaning of CPE has been widely ascribed to the surface roughness of solid electrodes, which causes an uneven arrangement of interfacial capacitances, current densities, etc., across the electrode surface [26]. This is consistent with the observed variety in the morphology and thickness of the surface layer formed during electrochemical processing. Another possible explanation of non-ideal Warburg behavior is the finite regime of diffusion within surface film (layer). Depending on the conditions (whether the finite-diffusion region is permeable/impermeable to the diffusing species), a transmissive or reflective boundary is established with the time, and as a consequence, EIS will exhibit a second semicircle or result in a (purely) capacitive behavior [27].

(2) The semicircle at high-frequency region is well pronounced for curves after 10 or 50 charge–discharge cycles. An increase in the time of electrochemical treatment eventually results in an almost complete disappearance of the semicircle at 500 cycles. It is known that the probability of the separation of the semicircle and Warburg straight line depends on the relationship between the R_ct_, Z_W_, and C_dl_ [27]. The model simulation shows that increasing C_dl_ while keeping R_ct_ and Z_W_ constant ultimately leads to the complete disappearance of the semicircle. In our case, etching the surface, leading to an increase in its specific area and the formation of layers on the surface, may well be the basis for a significant increase in the capacity C_dl_ and, accordingly, for the merging of the semicircle and Warburg straight line in the EIS curves.

(3) The point of expected intersection of the semicircle of the EIS plot with the real axis, which is defined by the sum of R_u_ + R_ct_, shifts substantially to the higher values with the number of cycles. This fact clearly indicates an increase in the charge-transfer resistance, R_ct_ (which is related to the kinetics of the heterogeneous electrochemical process). The nature of this effect remains unclear, but it can be assumed to be related to surface modification due to strontium leaching.

## 3. Materials and Methods

Polycrystalline ceramic cobaltite Sr_0.8_Dy_0.2_CoO_3−δ_ was synthesized from high-purity oxides Co_3_O_4_ (99.7%, metals-based, Alfa Aesar, Ward Hill, MA, USA), Dy_2_O_3_ (99.99%, REO, Alfa Aesar), and carbonate SrCO_3_ (99.99%, metals-based, Alfa Aesar) using a standard solid-state reaction. Stoichiometric amounts of the components were thoroughly mixed in an agate mortar with ethanol and, after drying, annealed at 1423 K for 16 h with intermediate grinding and calcination. The product was then subjected to high-energy wet grinding in ethanol using a Pulverisette 7 Premiumline planetary micromill (Fritsch GmbH, Germany, Idar-Oberstein) with a tungsten carbide grinding bowl and 3 mm grinding balls at a rotation speed of 500 rpm for 1 h. The alcohol was evaporated, and the dry sample was uniaxially pressed into tablets 12 mm in diameter and 1 mm thick under a pressure of 12 MPa. The tablets were then heated in air to 523 K at a rate of 50 °C/h, held at this temperature for 1 h, and then heated to 1473 K at a rate of 100 °C/h. The samples were held at 1473 K for 8 h and cooled to ambient temperature.

Powder X-ray diffraction (PXRD) data were acquired on a diffractometer PANalyticalX’PertPRO, (Almelo, The Netherlands) equipped with a PIXcel solid-state detector using CoKα radiation in the angle range 2θ 10–150 deg. Powder samples were prepared by octane grinding of the SDC bar in an agate mortar and packed in a flat sample holder for PXRD measurements in Bragg–Brentano geometry. Refinement of the full-profile PXRD was performed using the derivative difference minimization (DDM) method [28].

The morphology and local elemental composition of the samples were investigated using field-emission scanning electron microscopy (FE-SEM) and energy dispersive X-ray spectroscopy (EDS). The SEM and EDS experiments were performed with a JEOL JSM-7001F (Osaka, Japan) equipped with an energy dispersive X-ray spectrometer (Oxford Inca PentaFETx3).

The simultaneous thermal analysis (STA) experiments were carried out on a TG-DSC Netzch STA 449C analyzer (Germany, Selb) equipped with an AёoLOS QMS 403C mass spectrometer. The thermogravimetry (TG) and differential scanning calorimetry (DSC) measurements were performed in the dynamic O_2_-Ar atmosphere in platinum crucibles at a ramp rate of β = 10°/min. The thermal analysis was carried out on SDC powder with a 0.1–0.16 mm particle size and a sample mass of 30 mg.

**Figure 10:**
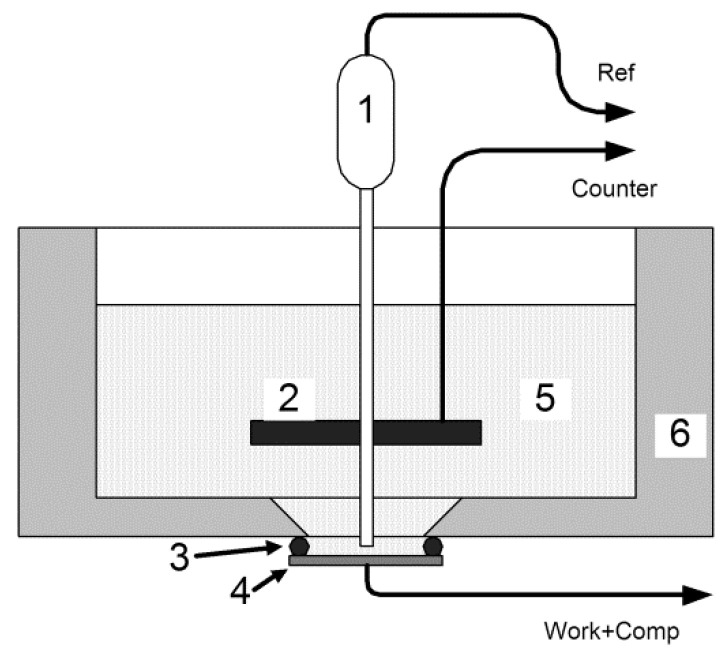
Design of an electrochemical cell. 1—Reference electrode (Ag/AgCl); 2—counter graphite disk electrode; 3—rubber sealing ring; 4—sample; 5—3M KOH solution; 6—PTFE housing.

The electrochemical performance of the SDC electrode was tested using cyclic voltammetry (CV), galvanostatic charge/discharge (GCD), and electrochemical impedance spectroscopy (EIS) on a three-electrode mode (Figure 10) at 298 K with a potentiostat-galvanostat P-45X, Electrochemical Instruments (Russia, Moscow). A dense SDC ceramic disk (12 mm diameter × 1 mm thickness), a graphite plate, and an Ag/AgCl electrode were used as the working, counter, and reference electrodes, respectively. All tests were carried out in a 3M KOH electrolyte. The CV measurements were performed at scan rates of 2, 5.3, 14, 37, and 100 mV/s. GCD tests were performed at current densities of 1 and 2 A/m^2^.

EIS data were obtained using an AC voltage of 10 mV in the frequency range of 0.01–10^5^ Hz. The specific capacitance (*C_sp_*) of the SDC electrode was estimated from the CV curves using Equation (9).

(9) $$C_{sp}=\frac{\left(I_{max}-I_{min}\right)}{2\nu S}$$

where *C_sp_* is the specific capacitance, F/m^2^; *I_max_, I_min_* is the current at given E (vs. Ag/AgCl) (A); ν is the scan rate, V/s; and *S* is the electrode area subjected electrochemical treatment in KOH solution (m^2^).

The working surface of electrode S was set to 8.17 × 10^−5^ m^2^, which is the surface area of the SDC disk in contact with the working solution. This surface area is limited by the size of the sealing rubber ring, which has a diameter of 10.2 mm. No corrections were applied for surface roughness or the porous structure of the ceramics, which can be estimated using published methods [29]. This approach’s validity is confirmed by the similarity of the capacitance values for pre-polished and unpolished electrodes, as well as the absence of visible traces of electrochemical action outside the rubber seal and on the back of the disk in contact with the conductive collector. This indicates the ceramic material’s lack of significant porosity.

## 4. Conclusions

The monolithic ceramic tetragonal nonstoichiometric dysprosium-doped strontium cobaltite Sr_0.8_Dy_0.2_CoO_3n−δ_ (SDC) demonstrated relatively high values of specific capacitance up to 500 F/m^2^.

The presence of mobile anion vacancies in the SDC structure is presumed to provide elevated values of specific capacity through the implementation of an intercalation mechanism involving O^2−^.

The SDC-based electrode showed good stability in a multi-cycle charge–discharge process (500 cycles). During electrochemical exposure, depletion of the electrode surface in strontium and the formation of a thin 90 nm film were observed. This film, upon prolonged treatment, was replaced by a 500 nm thick globular layer formed by particles 200–300 nm in size.

The chemical similarity of the processes of gas-phase oxygen evolution/addition and liquid-phase intercalation of O^2−^ anions in an alkaline solution allows us to consider the mobility of oxygen/anion vacancies in rare earth-doped perovskites as an indicator of the possibility of obtaining materials with high values of specific capacity.

## Data Availability

The raw data supporting the conclusions of this article will be made available by the authors on request.

## Abbreviations

The following abbreviations are used in this manuscript:

CPE	constant phase element
CV	cyclic voltammetry
EDS	energy-dispersive X-ray spectroscopy
EIS	electrochemical impedance spectroscopy
SDC	strontium-dysprosium cobaltite
SEM	scanning electron microscopy
RP	Ruddlesden−Popper
TG	thermogravimetry
XRD	X-ray diffraction
